# Implications of social distancing in Brazil in the COVID-19 pandemic

**DOI:** 10.1017/ice.2020.210

**Published:** 2020-05-08

**Authors:** Raquel Cristina Cavalcanti Dantas, Paola Amaral de Campos, Iara Rossi, Rosineide Marques Ribas

**Affiliations:** 1 Institute of Biotechnology, Federal University of Uberlandia, Brazil; 2 Laboratory of Molecular Microbiology, Federal University of Uberlandia, Brazil

*To the Editor*—The pandemic caused by a novel coronavirus disease, known as COVID-19, carried millions of people around the world to a state of unprecedented panic. The World Health Organization (WHO) stated that more than one-third of the world is currently under some social distancing pattern, which is the oldest and probably one of the most effective methods for controlling infectious disease outbreaks. However, governments of many countries have difficulty implementing social distancing, particularly in developing countries such as Brazil, where income inequality is high and the national economy is fragile.

Several studies in the literature, both in developed and developing countries, have demonstrated the effectiveness of social distancing in slowing the spread of COVID-19.^1,2^ In a recent study, Taghrir et al^1^ investigated the efficacy of mass quarantine during the pandemic and found good-quality evidence for the social distancing strategies to have been highly effective in controlling the spread of the disease. Complementing this analysis, other researchers analyzed data of 8 countries extremely affected by COVID-19: China, Italy, Iran, Germany, France, Spain, South Korea, and Japan. They concluded that the rapidly increasing COVID-19 case numbers in European countries occurs due to late contention measures^2^. Therefore, social distancing is currently the most effective way to slow the spread of COVID-19.

In Brazil, the Ministry of Health recommended measures of social distancing, respiratory etiquette, and hand hygiene.^3^ Social distancing measures included the closing of schools, universities, and almost all shops, except food stores and pharmacies. In addition, cafes, restaurants, clubs, gyms, museums, and other institutions across the country have closed. Public gatherings, religious services, and social and sporting events have been cancelled. Nonetheless, the number of cases for COVID-19 has continued to grow exponentially due to difficulties in establishing true and effective social distancing. In the real Brazilian context, a large number of informal workers are still working normally and there is a lack of access to information for a large part of the population regarding minimum infection prevention and control measures, including hand washing and respiratory etiquette.

Although handwashing and social distancing are still the best measures to protect against the virus, the flattening the COVID-19 curve will require additional measures in developing countries, where the spreading factor of the virus are different and more complex. In Brazil, it is essential to better understand the true prevalence of COVID-19, but the lack of mass testing is one of the main problems that make it difficult to implement measures to ensure that infected individuals are in an appropriate quarantine. Here, the physical distancing between infected and people is crucial in the high-risk group, such as the elderly and those with respiratory or chronic illnesses, to reduce the lethal effect of the pandemic.

According to the WHO, wearing a surgical mask, in combination with hand hygiene and other preventative measures, is one of the prevention measures to limit the spread of SARS-CoV-2 in affected areas.^4^ Cowling et al^5^ demonstrated that the implementation of social distancing measures and changes in population behaviors, including use of facial masks, were associated with reduced transmission of SARS-CoV-2 in Hong Kong. In Brazil, the adoption of this equipment can be difficult due to the low adhesion or the lack of access to facial masks by the Brazilian population. Thus, the correct use of facial masks is fundamental to the effectiveness of the measure and can be encouraged and improved through education campaigns.

In Brazil, coronavirus is advancing exponentially. Although the disease has spread rapidly in large capitals, where the incidence of cases is high, COVID-19 cases are increasing in smaller cities and poorer communities as well. More than three-quarters of the confirmed cases are in southern and southeastern regions of Brazil, which are more densely populated, including many elderly, and with tropical and subtropical climates. In addition, the economic burden that sustained distancing can impose is potentially catastrophic in Brazil and other developing countries. Furthermore, if social distancing is not effective and/or is not sustained for long enough, the healthcare system may collapse, contributing to a greater tragedy.
